# Combat high or traumatic stress: violent offending is associated with appetitive aggression but not with symptoms of traumatic stress

**DOI:** 10.3389/fpsyg.2014.01518

**Published:** 2015-01-07

**Authors:** Anke Köbach, Susanne Schaal, Thomas Elbert

**Affiliations:** ^1^Department of Psychology, University of KonstanzKonstanz, Germany; ^2^Vivo International (www.vivo.org); ^3^Department of Psychology, University of UlmUlm, Germany

**Keywords:** ex-combatant, combat high, demobilization, DDR, DR Congo, aggression, PTSD, violence

## Abstract

Former members of armed groups in eastern DR Congo had typically witnessed, experienced, and perpetrated extreme forms of violence. Enhanced trauma-related symptoms had been shown in prior research. But also lashing out in self-defense is a familiar response to threat defined as *reactive aggression*. Another potential response is *appetitive aggression*, in which the perpetration of excessive violence is perceived as pleasurable (combat high). What roles do these forms of aggressive behavior play in modern warfare and how are they related to posttraumatic stress symptoms? To answer the question, we sought to determine predictors for appetitive aggressive and trauma-related mental illness, and investigated the frequency of psychopathological symptoms for high- and low-intensity conflict demobilization settings. To this end, we interviewed 213 former members of (para)military groups in the eastern Democratic Republic of Congo in regard to their combat exposure, posttraumatic stress, appetitive aggression, depression, suicidality, and drug dependence. Random forest regression embedded in a conditional inference framework revealed that perpetrated violent acts are not necessarily stressful. In fact, the experience of violent acts that typically implicated salient cues of hunting (e.g., blood, suffering of the victim, etc.) had the strongest association with an appetite for aggression. Furthermore, the number of lifetime perpetrated violent acts was the most important predictor of appetitive aggression. However, the number of perpetrated violent acts did not significantly affect the posttraumatic stress. Greater intensity of conflict was associated with more severe posttraumatic stress symptoms and depression. Psychotherapeutic interventions that address appetitive aggression in addition to trauma-related mental illness, including drug dependence, therefore seem indispensible for a successful reintegration of those who fought in the current civil wars.

## Introduction

Exposure to combat has consistently been associated with heightened risks for posttraumatic stress disorder (PTSD) and other trauma-related symptoms, including substance use disorders and depression (Hoge et al., 2004; Dohrenwend et al., 2006; Odenwald et al., 2009; Schaal et al., 2012; Priebe et al., 2013; Heltemes et al., 2014). It has been argued that trauma symptoms in veterans are particularly prominent among those who have participated in killing (Macnair, 2002; Maguen et al., 2009, 2010, 2011; Van Winkle and Safer, 2011).

In addition to psychiatric disorders, a growing body of research reports elevated aggressive behaviors among (former) members of (para)military groups (Morland et al., 2012). Prior investigations have shown that violent outbursts (e.g., intimate partner violence; for a review, see Jones, 2012) are often associated with hypervigilance and/or impulsiveness that are typically related to trauma symptoms (Morland et al., 2012; MacManus et al., 2013). Moreover, there is growing evidence that active members of armed groups and ex-combatants may find violent acts appealing (Elbert et al., 2010, 2013; Weierstall et al., 2011, 2012a,b; Hecker et al., 2012, 2013; Haer et al., 2013). In fact, aggressive behavior usually is a mixture of the following forms (Vitiello and Stoff, 1997): reactive aggression describes an impulsive, affective and uncontrolled (automatic) violent behavior provoked by a perceived or real threat. Predatory aggression is a controlled action pursued to achieve a certain goal (Anderson and Bushman, 2002; Weierstall and Elbert, 2012). Appetitive aggression is motivated by intrinsic reward and thus describes the human potential to perceive perpetrated violence as fascinating and exciting (Elbert et al., 2010). Previous studies have revealed a positive relationship between the number of acts of violence an individual has perpetrated and appetitive aggression (Weierstall and Elbert, 2011; Weierstall et al., 2013b). Furthermore, a protective effect of appetitive aggression on posttraumatic stress symptoms has been noted in several war scenarios (Weierstall et al., 2011, 2012b, 2013a; Hecker et al., 2013). In addition to elevated interpersonal aggression, an increased risk-taking propensity has been reported among US army soldiers returning from Operation Iraqi Freedom (OIF; Killgore et al., 2008).

Combat exposure includes various forms of witnessed and experienced traumatic events as well as perpetrated violent acts that can be both traumatic and exciting or rewarding, respectively (Macnair, 2002; Elbert et al., 2010). To our knowledge, there is no research on the contribution of particular traumatic events and perpetrated acts to posttraumatic stress and appetitive aggression, yet. The high number of predictors, and/or the exclusion of potential confounding variables lead to weak statistical models with low external validity if parametric statistics would be used. Also relatively large clinical samples would be required and in many cases the models are not in accord with the theoretical basis of clinical psychology, which deals with *abnormal* behavior (i.e., behavior that is not typically found in the population and thus not normally distributed). Random forest with conditional inference trees (Hothorn et al., 2006b; Strobl et al., 2009a) is a flexible and attractive alternative. The non-parametric machine learning technique typically analyzes data sets with many predictors providing rankings according to their importance in regard to the factor. In the analysis we applied the method to examine the relation of different critical events/acts with appetitive aggression and posttraumatic stress, respectively.

In most post-conflict regions, the transition from war to peace is troubled with occasional fighting. The access to data concerning potentially varying levels of trauma-related symptoms in regard to the intensity of armed conflict is important to mental health care providers in these regions and therefore, will be addressed in this article.

We investigated demobilizing members of armed groups in North Kivu, where numerous foreign and local armed groups have been sustaining an extremely cruel armed conflict for two decades. Fear and violence are used not only to control territory and resources, but are also carried out in a seemingly self-perpetuating manner. Rape, torture and killing as well as atrocities such as cutting off ears, lips or breasts are frequent (Eck and Hultman, 2007). Soldiers witness, experience and commit extreme forms of violence in their everyday lives. To stabilize the region, MONUSCO (the United Nations Organization Stabilization Mission in the DRC) facilitates the demobilization of armed rebels. The mandate allows active soldiers to give up their weapons and uniforms and return to their home villages as civilians. The data assessment took place during two differing phases regarding the intensity of the conflict in the eastern Democratic Republic of Congo (DRC; LC phase = low-intensity conflict and HC phase = high-intensity conflict). In November 2012, the security and political situation in the DRC seriously deteriorated after a period of stability. At the peak of its territorial extension, the (at the time) most influential non-governmental armed group, the M23 (Movement Mars 23), captured the regional capital of Goma. Atrocities and insecurity increased until November 2013, when the M23 was defeated.

## Materials and methods

### Participants

All Congolese ex-combatants who joined the MONUSCO Disarmament, Demobilization and Reintegration (DDR) program during the study period and who were older than 18 years were interviewed. A total of 118 interviews were conducted during the LC phase, and 95 interviews were conducted during the HC phase. Table 1 shows the basic sociodemographic characteristics of the sample. The majority of participants belonged to the Hutu (49.3%, *n* = 105), Nande (20.2%, *n* = 43) or Hunde (12.7%, *n* = 27) ethnic groups. They had served as combatants for between 1 month and 24 years (*M* = 51.56, *SD* = 51.29), often for different armed groups (≥2 armed groups: 41.8%, *n* = 103). About two-thirds (65.4%, *n* = 140) reported that they had been forcibly recruited at least once, whereas 59.2% (*n* = 126) reported having voluntarily joined an armed group at least once. The mean age of first recruitment was 17.90 years (*SD* = 6.05, range: 4–35). More than two-thirds (67.5%, *n* = 143) of the ex-combatants were recruited for the first time before the age of 18 years. Participants had fought for as many as five armed groups (*M* = 1.84, *SD* = 1.08) and reported having demobilized from various local Mai-Mai groups (39.9%, *n* = 85), armed groups from Rwanda (i.e., Forces Démocratique pour la Libération du Rwanda, FDLR; 22.1%, *n* = 47), the Patriots Résistants Congolais (Pareco; 5.2%, *n* = 11) and the M23/CNDP (Movement 23/Congrés National pour la Defense du People; 30.0%, *n* = 64). The M23/CNDP was the most influential armed group at the time of the study. 2.8% (*n* = 6) of participants reported that they had fought for other armed groups. The participants did not significantly differ in their sociodemographic characteristics according to their group affiliation.

**Table 1:** **Sociodemographic data, exposure to violence, and mental health of combatants who demobilized in the low- and high-intensity conflict phases (LC and HC)**.

	**LC phase**		**HC phase**		
	**M ± SD**	**[CI]**	**M ± SD**	**[CI]**	***t*_(211)_**
**SOCIODEMOGRAPHIC DATA**					
Age in years	23.09 ± 4.93	[22.19–23.99]	24.35 ± 6.46	[23.04–25.67]	−1.62
Years of education	4.24 ± 4.17	[3.48–5.00]	5.00 ± 3.92	[4.20–5.80]	−1.36
Months spent in the AG	46.92 ± 47.33	[38.21–55.62]^a^	57.23 ± 55.47	[45.93–68.53]	−1.46^b^
**EXPOSURE TO VIOLENCE**					
Lifetime traumatic events (wit.)	5.53 ± 2.04	[5.16–5.91]	6.38 ± 1.32	[6.11–6.65]	−3.49^***^
Lifetime traumatic events (exp.)	4.34 ± 2.19	[3.94–4.74]	5.66 ± 1.85	[5.29–6.04]	−4.69^***^
Lifetime perpetrated violence	2.91 ± 2.38	[2.47–3.34]^c^	4.44 ± 1.80	[4.08–4.81]	−5.20^***^^d^
**MENTAL HEALTH**					
PSS-I total score	9.70 ± 8.67	[8.12–11.28]	15.42 ± 9.60	[13.47–17.38]	−4.56^***^
Facilitative Aggression (AFAS)	4.00 ± 6.70	[2.78–5.22]	–		−
Appetitive Aggression (AFAS)	2.95 ± 6.03	[1.85–4.05]	–		−
Appetitive Aggression (AAS)	–		21.91 ± 14.80	[18.89–24.92]	−
PHQ-9 total score	5.10 ± 5.19	[4.16–6.05]	7.40 ± 5.33	[6.31–8.49]	−3.17^**^
TCUDS II total score	2.59 ± 2.93	[2.06–3.13]	2.54 ± 3.05	[1.92–3.16]	0.14

^*^p < 0.05,

** *p < 0.01*,

*** *p < 0.001*;

a *n = 116*,

b *t_(209)_*;

c *n = 117*,

d *t_(210)_*;

*LC phase: n = 118; HC phase: n = 95; wit., witnessed; exp., experienced; AG, armed group; PSS-I, PTSD Symptom Scale-Interview; AFAS, Appetitive and Facilitative Aggression Scale; AAS, Appetitive Aggression Scale; PHQ-9, Patient Health Questionnaire-9, (depression); TCUDS II, Texas Christian University Drug Screen II*.

### Procedure

Interviews were conducted individually at a secluded place in the MONUSCO demobilization camp as part of the respective DDR program. Interviews lasted between 1.5 and 2.5 h. Participants gave their informed consent in writing or (if illiterate) verbally. All of the subjects who were approached agreed to participate. There was no financial compensation offered. All ex-combatants who arrived at the camp during the following time periods were interviewed and included in the analysis: July 23^*rd*^ to 29^*th*^, August 7^*th*^ to 17^*th*^ and August 30^*th*^ to September 15^*th*^, 2012 (LC phase); February 2^*nd*^ to 11^*th*^, February 27^*th*^ to March 13^*th*^ and March 26^*th*^ to April 5^*th*^, 2013 (HC phase). The ethical commission of the *University of Konstanz* approved the study. The questionnaires used in the study were translated into Kiswahili and back by independent groups of translators from Goma. The interviews were conducted by a group of local interviewers (one psychologist, four psychology students and one translator). These interviewers were trained during an intensive 10-day session in the basic theoretical concepts underlying the research and in sensitive and empathic interviewing techniques. The interviewers received follow-up training in October 2012 (3 days) and in February 2013 (7 days). Throughout the data collection periods, interviewers were closely supervised by clinical experts and received extensive feedback. All of the diagnostic instruments described in the following section were administered as clinical interviews.

### Measures

Sociodemographic information was obtained from each participant and included age, ethnicity, educational background and details regarding the participant's military career.

A 31-item event checklist adapted from previous studies of similar populations (Hecker et al., 2012, 2013) was administered to assess lifetime exposure to different types of potentially traumatic events (experienced and witnessed) and perpetrated violent acts (war and non-war related). The total number of types of witnessed (possible range: 0–10) and experienced (possible range: 0–12) traumatic events and the number of types of perpetrated violent acts (possible range: 0–9) was calculated. Reliability measures showed that the applied event checklist had good consistency (Cronbach's α = 0.87) and high inter-rater reliability (Cohen's κ = 0.89).

Participants' diagnostic status and PTSD symptom severity were assessed using the PTSD Symptom Scale-Interview (PSS-I; Foa and Tolin, 2000). The PSS-I assesses the 17 DSM-IV (APA, 1994) symptom criteria for PTSD and assesses symptom intensity during the previous month. Each item is rated on a four-point scale ranging from 0 (*not at all/only once*) to 3 (*five or more times per week/almost always*). PTSD severity was calculated by summing all of the symptom scores (possible scores range from 0 to 51). Internal consistency and inter-rater reliability revealed excellent values (Cronbach's α = 0.89; *ICC* = 0.98).

Appetitive aggression was assessed using the Appetitive and Facilitative Aggression Scale (AFAS; LC phase) and the Appetitive Aggression Scale (AAS, Weierstall and Elbert, 2011; HC phase). The AAS has been shown to have excellent psychometric properties but does not assess reactive aggression. The 30-item AFAS assesses current appetitive and reactive (“facilitative”) aggression during the four weeks prior to testing. Subjects rate statements on a five-point frequency scale ranging from 0 (*never*) to 4 (*more than two times a week*). Items include the following: “Was it fun for you to fight?;” “Did you look at violent imagery and find that you needed to see even more violent pictures to maintain your fascination?;” “Did you feel relieved after you screamed at someone?” The AFAS scores for reactive, appetitive and total aggression are calculated by adding the item scores (possible scores range from 0 to 60 for both reactive and appetitive aggression and from 0 to 120 for the total AFAS score). The psychometric properties for the total score were excellent (Cronbach's α = 0.94 and *ICC* = 0.89). The AAS consists of only 15 items, which are rated by respondents on a five-point scale ranging from 0 (*I totally disagree*) to 4 (*I totally agree*). The items solicit information about participants' appetitive perception of violence (e.g., “Is it exciting for if you make an opponent really suffer?;” “Once fighting has started, do you get carried away by the violence?”). The AAS has been successfully implemented (Hecker et al., 2012; Weierstall et al., 2012b) and validated (Weierstall and Elbert, 2012) in comparable East African samples. The AAS score is calculated by adding the scores of the 15 items (possible scores range from 0 to 60). Psychometric property measures indicated excellent internal consistency (Cronbach's α = 0.91) and high inter-rater reliability (*ICC* = 0.96) in this study.

Diagnostic status and depression symptom severity were determined using the Patient Health Questionnaire-9 (PHQ-9; Kroenke and Spitzer, 2002). The nine items correspond to the DSM-IV symptom criteria for major depression and assess the participants' feelings two weeks prior to testing. Each item is rated on a four-point scale ranging from 0 (*not at all*) to 3 (*nearly every day*). Following recommendations in the literature, cut-off values of 5, 10, 15, and 20 represent the respective thresholds for mild, moderate, moderately severe and severe depression (Löwe et al., 2004; Kroenke et al., 2010). In this sample, the Cronbach's α coefficient was.84, and the *intraclass correlation coefficient* indicated excellent inter-rater reliability (*ICC* = 0.96).

Drug dependency was diagnosed according to DSM-IV symptom criteria using the Texas Christian University Drug Screen II (TCUDS II; Knight et al., 2002), a standardized ten-item tool that assesses (yes/no) each criterion according to the participant's behavior in the past 12 months. In evaluations, the instrument has demonstrated stability across racial and ethnic subgroups (Simpson et al., 2012). Its psychometric properties are highly satisfactory (Cronbach's α = 0.90, Cohen's κ = 0.90).

### Analysis

The descriptive data are presented as frequencies (%), means and standard deviations. Group differences were analyzed using independent sample *t*-tests for continuous variables and *Chi^2^* tests for categorical variables. *Fisher's Exact* test was applied in cases of cell frequencies less than five. The reported statistical tests are two-tailed. Inter-rater interviews (*N* = 44) were conducted throughout the assessment process. Following Hallgren (2012), we computed *intraclass correlation coefficients* (*ICC*; two-way, mixed; absolute agreement) for ordinal data and *Cohen's Kappa (κ)* for nominal data using SPSS Version 21.

To consider complex interactions of the applied predictors (as suggested in recent research on appetitive aggression; (Weierstall et al., 2012b; Hecker et al., 2013) as well as to overcome limitations in sample size, non-linearity and homoscedasticity required for robust parametric analysis, we used a particular form of classical random forest (Breiman, 2001) embedded in a conditional inference framework (hereafter “conditional inference random forests” or RF-CI; Hothorn et al., 2006b). Unlike the classical random forest, the RF-CI does not display a bias toward predictors with many categories in the variable selection process (Strobl et al., 2008). Following the principles of ensemble methods, a certain number of trees (ntree) are aggregated to compose the random forest. Each tree is built using binary splits of the previously subsampled data (subsampling rate = 63.2%; Strobl, 2008). The splitting variable is chosen according to the strength of the association between the covariates and the outcome (Hothorn et al., 2006b; Strobl et al., 2009a) from a set of randomly preselected predictors (*p*, mtry, *p*/3; Grömping, 2009). Next, the importance of each predictor variable is ranked based on the ensemble of trees (conditional variable importance, *cvi*; Strobl et al., 2008). To visualize the results, we built single trees from the whole data set. These are, however, less robust (e.g., biased by outliers) and should not be interpreted without considering the results of the whole ensemble. The *goodness of fit* can be assessed using the out-of-bag data (OOB). The results are used to calculate a pseudo-*R^2^* from the mean squared error (MSE) and the total sum of squares (SST; *OOB-R^2^* = 1 - MSE/SST; Grömping, 2009).

To explore the relationship between appetitive aggression, posttraumatic stress and traumatic events/violent acts, we computed four separate RF-CI models using the data from the HC phase to assure comparability (*n* = 95). In the first two models, we regressed specific events/acts on appetitive aggression and posttraumatic stress levels and, in the remaining two models, on the number of traumatic events, separating witnessed and experienced traumatic stressors and violent acts.

The random forest analysis was conducted using *R* (version 2.15.0). The implementation we used was cforest (Hothorn et al., 2006b) from the *R* package party (Strobl et al., 2009b) with unbiased variable selection (Hothorn et al., 2006a,b). Details, including code and results for the four RF-CI models, can be accessed in the Supplemental Material of this article, which is available online.

## Results

### Exposure to violence

Combatants were exposed to a broad range of life-threatening experiences and reported having perpetrated various violent acts. This was particularly true among those who demobilized during the HC phase. These details are shown in Table 1. Table 2 lists the five most frequently reported experiences for each category.

**Table 2:** **Lifetime exposure to violence (witnessed, experienced, and perpetrated)**.

	**LC phase % (*n*)**	**HC phase % (*n*)**	*χ^2^*(1)
**WITNESSED**			
Have you ever witnessed dead bodies?	89.8 (106)	97.9 (93)	5.57^*^
Have you ever witnessed someone being physically assaulted (e.g., slapped)?	86.4 (102)	95.8 (91)	5.41^*^
Have you ever seen someone being killed (or killing him/herself)?	81.4 (96)	91.6 (87)	4.55^*^
Have you ever witnessed someone being physically assaulted with a weapon?	76.3 (90)	95.8 (91)	15.7^***^
Has a close friend/family member ever had a life-threatening illness or injury?	71.2 (84)	87.4 (83)	8.14^**^
**EXPERIENCED**			
Have you ever been assaulted with a weapon (e.g., shot, stabbed)?	73.7 (87)	91.6 (87)	11.21^**^
Have you ever been physically assaulted (e.g., slapped, kicked, beaten up)?	61.0 (72)	84.2 (80)	13.85^***^
Have you ever suffered from a life-threatening illness or injury?	67.8 (80)	74.7 (71)	1.23
Have you ever experienced a life-threatening explosion?	52.2 (62)	77.9 (74)	14.66
Have you ever been threatened to be killed by your commander/superior?	56.8 (67)	66.3 (63)	2.01
**SELF-COMMITTED**			
Have you ever stolen food to survive?	72.0 (85)	74.7 (71)	0.20
Have you ever you ever killed someone?	53.4 (63)	92.6 (88)	39.28^***^
Have you ever physically assaulted someone with a weapon (e.g., shot, slapped)?	38.1 (45)	86.3 (82)	50.75^***^
Have you ever physically assaulted someone (e.g., slapped, kicked, beaten up)?	39.8 (47)	70.5 (67)	19.93^***^
Have you ever attacked a village or settlement?	39.8 (47)	70.5 (67)	19.93^***^

*LC phase: n = 118; HC phase: n = 95*:

* *p < 0.05*,

** *p < 0.01*,

*** *p < 0.001*.

### Mental health and appetitive aggression

Table 1 summarizes the severity of PTSD, appetitive aggression, depression, and substance dependence for high (HC phase) and low (LC phase) conflict intensity.

#### PTSD

**Figure 1:**
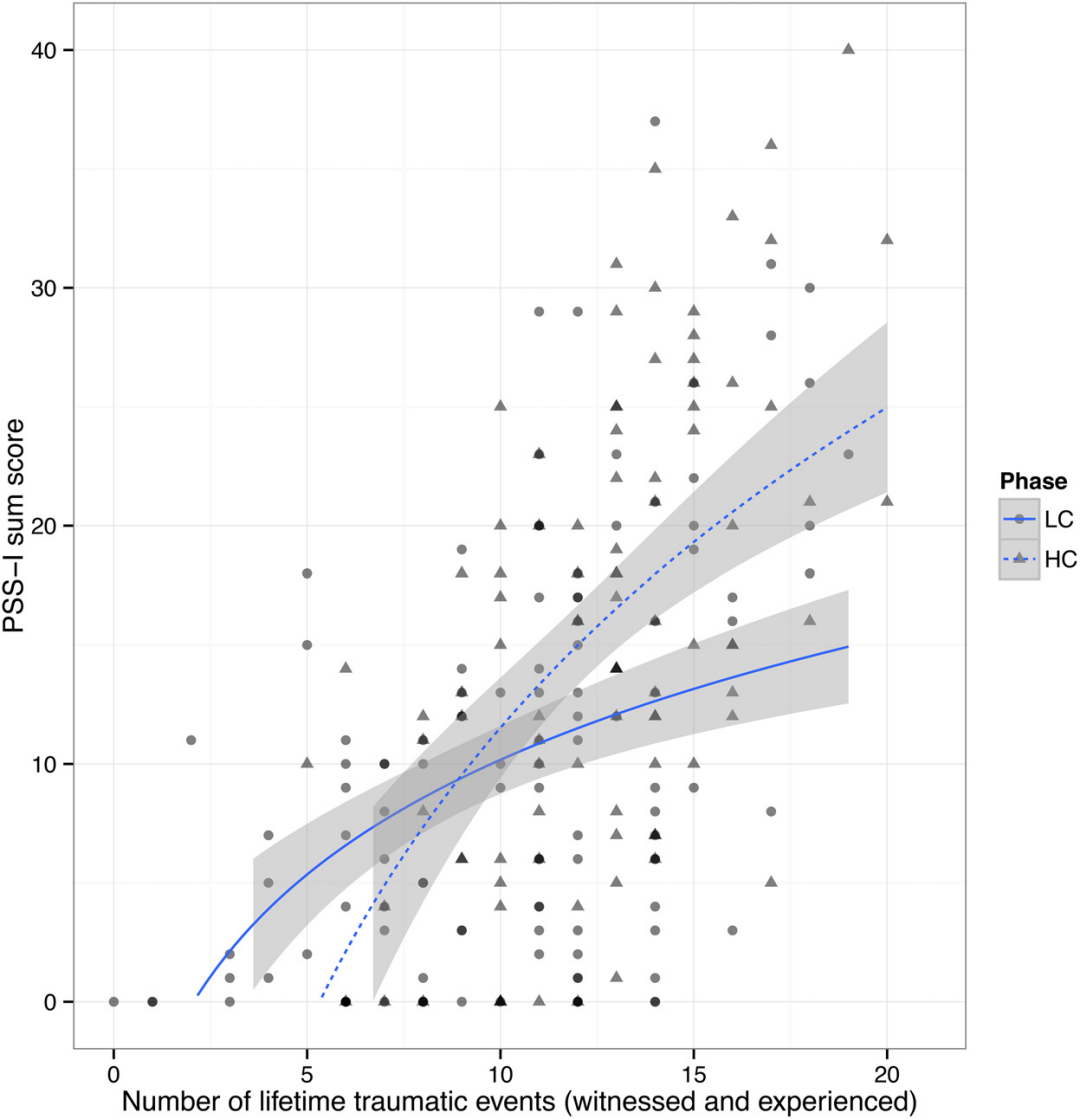
**The logarithmic least square fitted conditional mean (smoothed with 95% CI) of the PSS-I total score and the number of lifetime traumatic events by low-intensity and high-intensity conflict phase (LC and HC)**. Note: In addition to the sole effect of the higher number of traumatic events ex-combatants in the HC phase had experienced, conflict intensity seems to boost PTSD symptoms in ex-combatants with middle to high trauma load (<12 events).

A total of 32.7% (*n* = 70) of the participants fulfilled the diagnostic criteria for PTSD. The rate for current PTSD of ex-combatants interviewed during the HC phase was 44.2% (*n* = 42) compared to 23.5% (*n* = 28) for ex-combatants demobilizing during the LC phase, χ^2^(1, *N* = 213) = 10.27, *p* = 0.002. Figure 1 illustrates that conflict intensity boosts PTSD symptoms in ex-combatants with middle to high trauma load (<12 events), in addition to the sole effect of the higher number of traumatic events ex-combatants in the HC phase had experienced. The amount of year(s) since the worst events did not differ between the two phases [*M*_LC_ = 2.87, *SD*_LC_ = 3.41; *M*_HC_ = 2.78, *SD*_HC_ = 4.07; *t*_(208)_ = 0.22, *p* = 0.83].

#### Major depression, drug dependence, and suicidal ideation

Participants who demobilized in the high conflict setting (HC phase) suffered more from mild (HC: 29.5%; LC: 20.2%), moderate (HC: 23.2%; LC: 17.6%), and moderate to severe (HC: 12.3%; LC: 4.2%) depression than did those who demobilized during the LC phase, χ^2^(4, *N* = 213) = 12.47, *p* = 0.009. The DSM-IV diagnostic criteria for drug dependence were met by 41.6% (*n* = 89) of the total sample. There was no significant difference in the rate of drug dependence between the LC (42.0%, *n* = 50) and HC (41.1%, *n* = 39) phases, χ^2^(1, *N* = 213) = 0.02, *p* = 0.89. More than half of the participants who fulfilled the diagnostic criteria for PTSD also suffered from drug dependence (60.0%, *n* = 42). Low-level suicidal ideations were present in approximately a quarter of the participants (LC phase: 26.3%, *n* = 31; HC phase: 25.3%, *n* = 24). Between 5 and 10% reported a moderate (LC phase: 2.2%, *n* = 3; HC phase: 5.3%, *n* = 5) to high (LC phase: 5.9%, *n* = 7; HC phase: 9.5%, *n* = 9) suicide risk, χ^2^(3, *N* = 213) = 2.20, *p* = 0.54.

#### Appetitive aggression

Table 1 reports the level of appetitive (and facilitative) aggression for the LC and HC phases. In the HC phase, the AAS items reveal high levels of appetitive aggression: about 73.7% (*n* = 70) confirm that having defeated a strong opponent made the fight more pleasurable in comparison to the defeat of a weak opponent, and half of the respondents agreed that, while fighting, they had stopped caring about being killed (48.4%, *n* = 46). About a half of the respondents also stated that it had been exciting to make the opponent really suffer (48.4%, *n* = 46), that the desire to kill had taken control over them (46.3%, *n* = 44) and that they had felt powerful when they went to fight (44.2%, *n* = 42). For about a third of participants in the HC phase, it had been fun to prepare for fighting (34.7%, *n* = 33), they had experienced getting carried away by the violence (30.5%, *n* = 29) and seeing the victims' blood had made the fighting even more enjoyable (29.5%, *n* = 28). About a quarter had experienced the “thirst” to fight (23.2%, *n* = 22), reported a habituation to cruelty (23.2%, *n* = 22) and said that they had enjoyed listening to other people telling stories of how they killed (23.2%, *n* = 22). About a fifth (18.9%, *n* = 18) had enjoyed inciting their comrades to fight. A small group stated that they had harmed others just because they wanted to (9.5%, *n* = 9), that fighting is the only thing they had wanted to do in life (7.4%, *n* = 7) and that attacking humans had been sexually arousing (5.3%, *n* = 5).

### Prediction of appetitive aggression and posttraumatic stress by lifetime traumatic events and perpetrated acts

#### Specific events (witnessed, experienced, and perpetrated)

**Figure 2:**
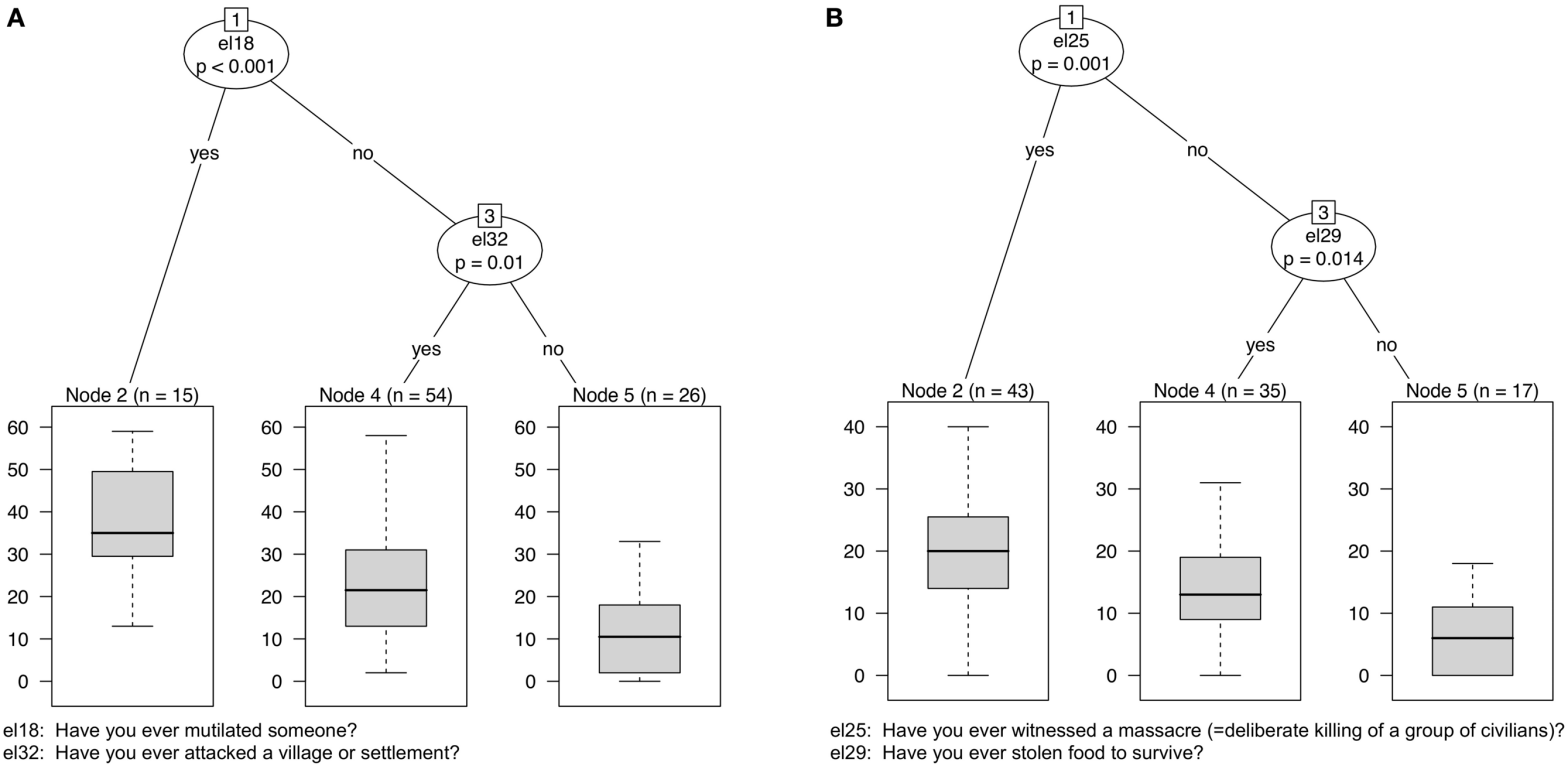
**Single trees for predicting AAS and PSS-I scores by specific events**. Note: Regression trees with asymmetric interactions for the appetitive aggression level (AAS score; **A**) and PTSD severity (PSS-I score; **B**) predicted by 31 items. **(A)** Significant partitioning is primarily achieved by the item “*Have you ever mutilated someone?*” For those who answered this question in the negative, “*Have you ever attacked a village or settlement?*” provides further significant partitioning. The box plots present the means, quartiles and ranges for the AAS scores in each group. Those having mutilated someone scored highest on the AAS, whereas those having neither mutilated someone nor attacked a village or settlement had the lowest AAS scores. Ex-combatants who did not mutilate someone but had attacked a village or settlement show the widest range. **(B)** The PSS-I score is first split by the item “*Have you ever witnessed a massacre?*” and partitions best for those who answered the item “*Have you ever stolen food to survive?*” in the negative.

The five incidents that best predicted the AAS score were as follows: “mutilating another person” (*cvi* = 16.44), “witnessing massacre” (*cvi* = 9.06), “attacking villages/settlements” (*cvi = 8.30)*, “assaulting someone physically” (*cvi* = 7.51), “participating in massacre” (*cvi* = 6.52) and “witnessing a sexual assault” (*cvi* = 4.79). These factors explained 33% of the variance in the out-of-bag data. After building a single tree from the whole data set, “mutilating another person” and “attacking villages/settlements” emerged as the variables with the highest impact on participants' level of appetitive aggression (see Figure 2A).

The five most important predictors for posttraumatic stress were as follows: “*witnessing massacre*” (*cvi* = 7.19), “*participating in massacre*” (*cvi* = 5.67), “*stealing food to survive*” (*cvi* = 2.10), “*witnessing a sexual assault*” (*cvi* = 1.71) and “*being threatened by the commander*” (*cvi* = 1.15). The model explained 27% of the variance in the out-of-bag data. The Supplemental Material (available online) contains the conditional variable importance of each specific event to the prediction of the AAS and PSS-I scores.

#### Number of lifetime events/acts (witnessed, experienced, and perpetrated)

*Perpetrated violent acts* had the highest predictive value for appetitive aggression (*cvi* = 87.57), compared to *experienced* (*cvi* = 3.72) and *witnessed* (*cvi* = 3.67) *traumatic events*. *Experienced* (*cvi* = 10.33) and *witnessed* (*cvi* = 9.25) *traumatic events* had the highest impact on participants' PSS-I score. The importance of the number of lifetime *perpetrated violent acts* in predicting the PSS-I score was *cvi* = 1.97. The OBB-*R^2^* explained 33% of the variance for posttraumatic stress and 44% for appetitive aggression.

By comparing the variance explained (OBB-*R^2^*) by specific events to the number of traumatic events/violent acts regressed on PSS-I and AAS scores, better model fits were obtained for the accumulated lifetime events/acts. The total scores of lifetime experienced/witnessed events and perpetrated violent acts explained an additional 7% of the variance for posttraumatic stress and an additional 11% for appetitive aggression, compared to the variance explanations of specific events.

## Discussion

The main goal of the present study was to examine the association between traumatic and perpetrated events and appetitive aggression in a sample of demobilizing combatants. Random forest with conditional inference trees was applied to regress specific events (experienced, witnessed, and perpetrated) as well as the sum of these events on the appetitive aggression level and posttraumatic stress. The results primarily revealed that certain specific events and the total number of traumatic events/violent acts predicted both appetitive aggression and posttraumatic stress. The models that used the total scores of events/acts as predictors showed better fits for the two outcome variables. The number of perpetrated violent acts was the best predictor of appetitive aggression, while experienced traumatic events was the best predictor of posttraumatic stress.

Additionally, we compared the occurrence of trauma-related symptoms in phases of low- and high-intensity conflict (LC vs. HC phase). As expected, PTSD severity increased in the HC phase. Importantly, the actual increase in fighting may not be the causal mechanism behind the observed differences. However, the observation is relevant for practical reasons, helping aid workers meet the needs of former combatants and allowing local institutions to prepare for the varying severity of the trauma-related problems (including PTSD and subclinical depression) that arise with heightened conflict intensity.

Heightened levels of aggression have been found in military personnel exposed to torture, rape, killing and other atrocities (Weierstall and Elbert, 2011; Hecker et al., 2012; Morland et al., 2012; MacManus et al., 2013). In these cases, reactive aggressive behavior can be explained by the hypervigilance symptoms of (combat-related) PTSD (Morland et al., 2012). No attempt has yet been made to account for reports of combat high or to etiologically explain enhanced appetitive aggression that considers violence appealing and intrinsically motivating. Such an explanation, however, would be necessary to design interventions that help ex-combatants to reintegrate into society. The present study examined the extent to which experienced and witnessed traumatic events and perpetrated violent acts impact appetitive aggression. RF-CIs for predicting appetitive aggression was first conducted on specific events and then on the total number of event types experienced during one's lifetime. In accordance with Weierstall et al. (2011, 2013b) we found that experiencing a greater number of perpetrated violent acts was associated with higher levels of appetitive aggression. In fact, the accumulation of perpetrated violent acts was found to be the most important predictor of perceiving self-committed atrocities as appealing. Of much less significance was the number of witnessed and experienced traumatic events. By comparing the explained variances (specific and cumulative events), a better fit to the number of events was found. The result suggests a strong incident-symptom relationship for appetitive aggression that is analogous to the building block effect for PTSD (Mollica et al., 1998; Neuner et al., 2004; Kolassa and Elbert, 2007). Regarding the single tree built to predict the level of appetitive aggression, “*mutilating another person*” and “*attacking a village/settlement*”—both acts of violence—emerged as items with a major impact. Comprising cues such as blood, the suffering of the victim, screams and pursuing the victim, these two behaviors mimic the idea of hunting (Elbert et al., 2010).

A major cleft in the literature exists with regard on how to treat violent perpetrators. Is perpetration, as suggested by proponents of the Perpetration-Induced Traumatic Stress-concept (PITS; Macnair, 2002), itself a potentially traumatizing event that thus puts the perpetrator at increased risk for developing PTSD (Maguen et al., 2009, 2010, 2011; Komarovskaya et al., 2011)? Or can violence at times be appealing and may heightened levels of aggression even protect perpetrators from developing symptoms of traumatic stress (Weierstall et al., 2011, 2012a,b, 2013a; Hecker et al., 2013)? The present data analyses suggest that perpetrating violent acts does not significantly affect posttraumatic stress (see Figure 2). By contrast, experienced and witnessed traumatic events have a well-known substantial impact on posttraumatic stress. As seen in the regression trees for the total scores (presented in the Supplemental Material) the number of perpetrated violent acts does not, however, significantly contribute to the prediction of posttraumatic stress. Of the specific events analyzed, “*witnessing a massacre*” was the most significant predictor of posttraumatic stress, followed by “*participating in a massacre*.” However, “*having killed another person*” was not predictive of posttraumatic stress. In conclusion, these results demonstrate that the perpetration of violent acts is not necessarily traumatic and that, instead, the extent of exposure to varying traumatic stressors plays a crucial role in the development of PTSD.

The result can be explained by bipolar neural networks. The fear network—the associative mnemonic representation of stimuli constituting traumatic events—is a well-established etiological model for PTSD (Foa and Kozak, 1986; Elbert et al., 2011). Further, Elbert et al. (2010) postulate a second network incorporating stimuli that constitute perpetrated violent acts—the so-called hunting network. Both, traumatic stress and combat high require a high degree of arousal. The major difference between the two incidents is their valence: traumatic stress is perceived as fearful and aversive and combat high is perceived as appealing, lust-evoking and exciting. However, they often consist of similar cues. For instance, blood, screaming, gunfire, etc. may occur in traumatic events and/or perpetrated acts. The opposing valence of these bipolar networks prevents their fusion (Elbert et al., 2010). Hence, shared cues may fluidly be dominated either by the positive pole and thus incorporated into the hunting network or by a negative valence, which is associated with the fear network. Depending on the perceived valence, a particular cue (e.g., blood) will trigger a specific emotional state and consequently prime either approaching or avoiding behavior patterns (Elbert et al., 2010).

In this study, we applied random forest regression to investigate the association of appetitive aggression and posttraumatic stress with various traumatic event types and self-committed violent acts. This non-parametric method has remarkable potential for the statistical approach of common problems in clinical psychology. In our case, the ensemble method resolved the high number of specific events/acts applied as predictors and the violation of assumptions for linear regression. RF-CI provided new insight into the role of specific incidents in the development of appetitive aggression and posttraumatic stress without *a priori* exclusion of variables. Furthermore, random forest makes it possible to compare the impact of specific events/acts with the total number of these experiences, though not with a statistical significance measure. The results revealed that the model provided a better fit when posttraumatic stress was predicted by the total number of events/acts (not by specific events) as assumed by the building block effect (the more events, the higher the PTSD severity; Kolassa and Elbert, 2007; Schauer et al., 2003). This finding contributes to the confirmation of a polytraumatic approach to PTSD therapy, as realized, for example, in Narrative Exposure Therapy (NET; Schauer et al., 2011).

The results further indicated that PTSD severity differs according to the level of ongoing conflict intensity. Approximately 24% of respondents in the LC phase were diagnosed with PTSD, whereas a sizeable 44% of respondents in the HC phase fulfilled the DSM-IV's PTSD criteria. Observing the association of the number of witnessed and experienced events according to the LC vs. HC phase reveals that conflict intensity increases PTSD symptoms especially for those who exhibit a moderate to high trauma load (<12 traumatic event types). Moreover, depression symptom severity is also higher in the HC phase. By contrast, drug dependence (41.6%, *n* = 89) is common among former combatants irrespective of the ongoing conflict intensity.

The reported levels of appetitive aggression in the present study (HC phase) correspond with previous investigations of ex-combatants in the eastern DRC (Hecker et al., 2013, *M* = 27.7, *SD* = 13.70 and Weierstall et al., 2011; *M* = 21.5, *SD* = 8.1) and in other countries (Colombia: Weierstall et al., 2013a; *M* = 30.8, *SD* = 7.9). Although they had recently decided to demobilize, approximately half of the participants confirmed that it was exciting for them to make their opponent suffer and that hunting or killing could take control of them. A minority even stated that fighting is the major content in their lives. In contrast to a psychopathic behavior (emotionally cold, purely instrumental), acts of appetitive aggression have been described advantageous to survive in a hostile environment (Crombach et al., 2013; Weierstall et al., 2013b). It is important to note that this is still highly prevalent—at least during the demobilization process before the former fighters return into civil society.

In conclusion, trauma symptoms and heightened levels of aggression are common among demobilizing combatants, and psychotherapeutic interventions are therefore of major importance for a successful reintegration process. Elbert et al. (2012) developed Narrative Exposure Therapy for Forensic Offenders Rehabilitation (FORNET) to reduce symptoms of PTSD and appetitive aggression. In a randomized controlled trial conducted on a sample of former child soldiers in DRC (Hermenau et al., 2013a) observed that FORNET successfully decreased PTSD symptoms and facilitated reintegration by decreasing closeness to military life. Crombach and Elbert's (2014) results demonstrated that FORNET reduces the number of criminal acts committed by former street children in Burundi.

The present study has several limitations. Although all of the former combatants demobilizing through DDR in Goma during a given period were included in the study, our sample is not necessarily representative. First, not everyone who leaves an armed group demobilizes in a formalized way passing through MONUSCO. As they may be afraid of consequences, combatants with higher levels of appetitive aggression may leave or escape form their group and continue life either in their former context or at any other place. Second, different groups may demobilize in different ways during a given period. Owing to the cross-sectional and retrospective nature of the design, drawing conclusions about the causal or temporal relationships between the variables should be done cautiously.

## Conclusion

To interrupt the cycle of violence and establish peace in war-torn countries, we need to understand how cruel scenarios affect the behavior and mental health of survivors. The results of this study showed that the number of perpetrated acts, and especially the act of mutilating someone, contributes significantly to the rewarding processing of otherwise traumatic stressors. However, witnessing a massacre seems to be the most devastating to one's mental health. These effects may especially induce those who actively participated in atrocities to return to war (Hermenau et al., 2013b). To reduce re-recruitment, delinquency or domestic violence after demobilization, psychotherapeutic interventions that address both appetitive aggression and traumatic stress may be required during the reintegration process.

### Conflict of interest statement

The authors declare that the research was conducted in the absence of any commercial or financial relationships that could be construed as a potential conflict of interest.
