# Supplementary material for: Combat high or traumatic stress: violent offending is associated with appetitive aggression but not with symptoms of traumatic stress
Source: Front Psychol. 2015 Jan 7;5:1518. doi: 10.3389/fpsyg.2014.01518 (PMC4285743; doi:10.3389/fpsyg.2014.01518)
Supplement: Supplementary file 1 [file Presentation1.ZIP › RF-CI.R/RF-CI2.PTSD.spec.html]

Supplemental Online Material: PTSD and specific events


Combat high or traumatic stress: violent offending is associated with appetitive aggression but not with symptoms of traumatic stress

Corresponding author: Anke Köbach, University of Konstanz, Department of Psychology, Universitätsstrasse 10, 78467 Konstanz, Germany. E-mail: anke.koebach@uni-konstanz.de;
Konstanz, June 26th, 2014

# Supplemental Online Material: PTSD and specific events

Random forest - conditional inference (RF-CI)

RF-CI: regressing the specific events/acts on posttraumatic stress (PSS-I)

Lifetime exposure to violence

> el1: Have you ever been hit (with or without belt, board, stick etc) by one of your parents/your caretaker in a way that marks were left on your body?
>
> el2: Have you ever witnessed a family member being hit (with or without stick, board, belt, etc) by one of your other family members in a way that marks were left on his/her body?
>
> el3: Has one of your parents/your caretaker ever burnt your on purpose (e.g. hot water, cigarette, fire)?
>
> el4: Have you ever witnessed how one of your parents/your caretaker ever burnt your on purpose (e.g. hot water, cigarette, fire)?
>
> el5: Have you ever experienced a natural disaster (for example, flood, land side , volcano outbreak, earthquake) in such a way that your life was in danger?
>
> el6: Have you ever ecperienced a life-threatening fire or explosion?
>
> el7: Have you ever experienced an accident (e.g. car accident, bus accident, serious accident at work, home or, during recreational activity)?
>
> el8: Have you ever witnessed an accident (e.g. car accident, bus accident, serious accident at work, home or, during recreational activity)?
>
> el9: Have you ever suffered from a life-threatening illness or injury?
>
> el10: Has a close friend or family member ever had a life-threatening illness or injury?
>
> el11: Have you ever been physically assaulted (for example being attacked, hit, slapped, kicked, beaten up (includes beatings with sticks) in such a way that you had fear for your life?
>
> el12: Have you ever witnessed sombody being physically assaulted (for example being attacked, hit, slapped, kicked, beaten up (includes beatings with sticks)?
>
> el13: Have you yourself ever physically assaulted someone in such a way?
>
> el15: Have you ever been assaulted with a weapon (for example being shot, mutilated, stabbed, threatened with a knife, gun)?
>
> el16: Have you ever witnessed someone else being assaulted with a weapon (for example being shot, mutilated, stabbed, threatened with a knife, gun)?
>
> el17: Have you yourself ever physically assaulted someone with a weapon?
>
> el18: Have you ever mutilated another person with a weapon?
>
> el19: Have you ever seen somebody being killed or killing him-/herself?
>
> el20: Have you ever killed someone?
>
> el21: Have you ever experienced a sexual assault?
>
> el22: Have you ever witnessed a sexual assault?
>
> el23: Have you yourself ever sexually assaulted someone?
>
> el24: Have you ever witnessed dead bodies?
>
> el25: Have you ever witnessed a massacre (=deliberate killing of a group of civilians)?
>
> el26: Have you ever participated in a massacre?
>
> el27: Have you ever been threatened to be killed by your commander (e.g. for no reason, for disobeying rules, for failed escapeattempt)?
>
> el29: Have you ever stolen food to survive? (assigned to perpetrator events)
>
> el30: Have you ever eaten human flesh?
>
> el31: Have you ever been forced to eat human flesh?
>
> el32: Have you ever attacked a village or settlement?
>
> el\_other: Have you ever experienced any other frightening event not yet mentioned?

```
library(party)

attach(data_RF)

# Compute 500 trees from 10 randomly preselected predicotrs adopting
# unbiased variable selection

set.seed(124)

forest2 <- cforest(as.numeric(ptsd_ss) ~ el1 + el2 + el3 + el4 + el5 + el6 + 
    el7 + el8 + el9 + el10 + el11 + el12 + el13 + el15 + el16 + el17 + el18 + 
    el19 + el20 + el21 + el22 + el23 + el24 + el25 + el26 + el27 + el29 + el30 + 
    el31 + el32 + el_other, data = data_RF, controls = cforest_unbiased(mtry = 10, 
    ntree = 500))

# Compute conditional variable importance

vic2 <- varimp(forest2, conditional = TRUE)

write.table(vic2)
```

```
"x"
"el1" -0.0360204360570375
"el2" -0.092090078172776
"el3" 0
"el4" 0.303350335028502
"el5" 0.754852034481702
"el6" -0.0303088916358163
"el7" -0.146940911648896
"el8" 0.00654558597632601
"el9" 0.334849458936891
"el10" 0.497429802987009
"el11" 1.06336013081683
"el12" 0
"el13" -0.0105260415435793
"el15" -0.00376470588235296
"el16" 0
"el17" -0.0946628339749447
"el18" 0.208650145285675
"el19" 0
"el20" 0
"el21" 0.530050232470798
"el22" 1.70511044948075
"el23" -0.0666348558246828
"el24" 0
"el25" 7.19376790454828
"el26" 5.67221044588756
"el27" 1.14545442295453
"el29" 2.09898390142242
"el30" 0
"el31" 0
"el32" -0.108990377969723
"el_other" -0.0931692596344908
```

```
# Compute pseudo-Rsquared from the out-of-bag-data

pred2 <- predict(forest2, OOB = TRUE)

MSE2 <- mean((data_RF$ptsd_ss - predict(forest2))^2)
SST2 <- mean((data_RF$ptsd_ss - mean(data_RF$ptsd_ss))^2)
R_Sq2 <- (1 - (MSE2/SST2))

detach(data_RF)
```

MSE2=66.16

SST2=91.23

R\_SQ2=0.2748

```
attach(data_RF)


# png('PTSDspecevents.png', height=7, width=7, res=500, units='in') #store
# as pdf file

set.seed(124)

regress2tree <- ctree(as.numeric(ptsd_ss) ~ el1 + el2 + el3 + el4 + el5 + el6 + 
    el7 + el8 + el9 + el10 + el11 + el12 + el13 + el15 + el16 + el17 + el18 + 
    el19 + el20 + el21 + el22 + el23 + el24 + el25 + el27 + el29 + el30 + el31 + 
    el32 + el_other, data = data_RF, controls = ctree_control())

plot(regress2tree)
```

```
# dev.off()


detach(data_RF)
```
