# Supplementary material for: Combat high or traumatic stress: violent offending is associated with appetitive aggression but not with symptoms of traumatic stress
Source: Front Psychol. 2015 Jan 7;5:1518. doi: 10.3389/fpsyg.2014.01518 (PMC4285743; doi:10.3389/fpsyg.2014.01518)
Supplement: Supplementary file 1 [file Presentation1.ZIP › RF-CI.R/RF-CI3.AAS.sum.html]

Supplemental Online Material: AAS and the sum of events


Combat high or traumatic stress: violent offending is associated with appetitive aggression but not with symptoms of traumatic stress

Corresponding author: Anke Köbach, University of Konstanz, Department of Psychology, Universitätsstrasse 10, 78467 Konstanz, Germany. E-mail: anke.koebach@uni-konstanz.de; Konstanz, June 26th, 2014

# Supplemental Online Material: AAS and the sum of events

Random forest - conditional inference (RF-CI)

RF-CI: regressing the sum of events (witnessed, experienced and perpetrated) on the level of appetitive aggression

Lifetime exposure to violence

> el\_w: lifetime traumatic events - witnessed
>
> el\_e: lifetime traumatic events - experienced
>
> el\_p: lifetime perpetrated violent acts

```
library(party)

attach(data_RF)

# Compute 500 trees from 2 randomly preselected predicotrs adopting
# unbiased variable selection

set.seed(524)

forest3 <- cforest(as.numeric(aas_ss) ~ el_w + el_e + el_p, data = data_RF, 
    controls = cforest_unbiased(mtry = 2, ntree = 500))

# Compute conditional variable importance

vic3 <- varimp(forest3, conditional = TRUE)

write.table(vic3)
```

```
"x"
"el_w" 3.66952282966937
"el_e" 3.71874916982995
"el_p" 87.5705411861065
```

```
# Compute pseudo-R^2 from the out-of-bag-data

pred3 <- predict(forest3, OOB = TRUE)

MSE3 <- mean((data_RF$aas_ss - predict(forest3))^2)
SST3 <- mean((data_RF$aas_ss - mean(data_RF$aas_ss))^2)
R_Sq3 <- (1 - (MSE3/SST3))

detach(data_RF)
```

MSE3=121.77

SST3=216.63

R\_SQ3=0.4379

```
attach(data_RF)

set.seed(524)

regress3tree <- ctree(as.numeric(aas_ss) ~ el_e + el_w + el_p, data = data_RF, 
    controls = ctree_control())

plot(regress3tree)
```

```
detach(data_RF)
```
