# Supplementary material for: Combat high or traumatic stress: violent offending is associated with appetitive aggression but not with symptoms of traumatic stress
Source: Front Psychol. 2015 Jan 7;5:1518. doi: 10.3389/fpsyg.2014.01518 (PMC4285743; doi:10.3389/fpsyg.2014.01518)
Supplement: Supplementary file 1 [file Presentation1.ZIP › RF-CI.R/RF-CI4.PTSD.sum.html]

Supplemental Online Material: PTSD and the sum of events


Combat high or traumatic stress: violent offending is associated with appetitive aggression but not with symptoms of traumatic stress

Corresponding author: Anke Köbach, University of Konstanz, Department of Psychology, Universitätsstrasse 10, 78467 Konstanz, Germany. E-mail: anke.koebach@uni-konstanz.de;
Konstanz, June 26th, 2014

# Supplemental Online Material: PTSD and the sum of events

Random forest - conditional inference (RF-CI)

RF-CI: regressing the sum of events (witnessed, experienced and perpetrated) on posttraumatic stress (PSS-I)

Lifetime exposure to violence

> el\_w: lifetime traumatic events - witnessed
>
> el\_e: lifetime traumatic events - experienced
>
> el\_p: lifetime perpetrated violent acts

```
library(party)

attach(data_RF)

# Compute 500 trees from 2 randomly preselected predicotrs adopting
# unbiased variable selection

set.seed(124)

forest4 <- cforest(as.numeric(ptsd_ss) ~ el_e + el_w + el_p, data = data_RF, 
    controls = cforest_unbiased(mtry = 2, ntree = 500))


# Compute conditional variable importance

vic4 <- varimp(forest4, conditional = TRUE)

write.table(vic4)
```

```
"x"
"el_e" 10.3788915753316
"el_w" 8.48475571678772
"el_p" 1.8922371460891
```

```
# Compute pseudo-R^2 from the out-of-bag-data

pred4 <- predict(forest4, OOB = TRUE)

MSE4 <- mean((data_RF$ptsd_ss - predict(forest4))^2)
SST4 <- mean((data_RF$ptsd_ss - mean(data_RF$ptsd_ss))^2)
R_Sq4 <- (1 - (MSE4/SST4))

detach(data_RF)
```

MSE4=59.86

SST4=91.23

R\_SQ4=0.3439

```
attach(data_RF)

# png('PTSDspecevents.png', height=7, width=7, res=500, units='in')


set.seed(124)

regress4tree <- ctree(as.numeric(ptsd_ss) ~ el_e + el_w + el_p, data = data_RF, 
    controls = ctree_control())

plot(regress4tree)
```

```
# dev.off()

detach(data_RF)
```
